# Strategies to Improve Management of Acute Watery Diarrhea during a Military Deployment: A Cost Effectiveness Analysis

**DOI:** 10.4269/ajtmh.17-0196

**Published:** 2017-09-25

**Authors:** Andrew J. Schrader, David R. Tribble, Mark S. Riddle

**Affiliations:** 1 Uniformed Services University of the Health Sciences, Bethesda, Maryland;; 2 Naval Medical Research Center, Silver Spring, Maryland

## Abstract

To inform policy and decision makers, a cost-effectiveness model was developed to predict the cost-effectiveness of implementing two hypothetical management strategies separately and concurrently on the mitigation of deployment-associated travelers’ diarrhea (TD) burden. The first management strategy aimed to increase the likelihood that a deployed service member with TD will seek medical care earlier in the disease course compared with current patterns; the second strategy aimed to optimize provider treatment practices through the implementation of a Department of Defense Clinical Practice Guideline. Outcome measures selected to compare management strategies were duty days lost averted (DDL-averted) and a cost effectiveness ratio (CER) of cost per DDL-averted (USD/DDL-averted). Increasing health care and by seeking it more often and earlier in the disease course as a stand-alone management strategy produced more DDL (worse) than the base case (up to 8,898 DDL-gained per year) at an increased cost to the Department of Defense (CER $193). Increasing provider use of an optimal evidence-based treatment algorithm through Clinical Practice Guidelines prevented 5,299 DDL per year with overall cost savings (CER −$74). A combination of both strategies produced the greatest gain in DDL-averted (6,887) with a modest cost increase (CER $118). The application of this model demonstrates that changes in TD management during deployment can be implemented to reduce DDL with likely favorable impacts on mission capability and individual health readiness. The hypothetical combination strategy evaluated prevents the most DDL compared with current practice and is associated with a modest cost increase.

## INTRODUCTION

Acute watery diarrhea (AWD) has been a significant problem for militaries across the world for centuries. From General George Washington to Napoleon I to Erwin Rommel, military commanders have been plagued by reduced troop strength because of diarrhea, and it has been described to contribute to lost battles.^1^ When it occurs in travelers, it is generally referred to as travelers’ diarrhea (TD). In modern times, AWD continues to affect military service men and women when deployed to developing countries.^2^ There can be serious ramifications to a military mission if even just a handful of a unit’s troop strength is rendered nonmission capable for even a day.

TD can be caused by one of several enteric pathogens; most often the etiologic pathogen is bacterial (> 80%).^3^ It is characterized by the development of AWD, dysentery, fever, or acute gastroenteritis with enterotoxigenic *Escherichia coli*, *Campylobacter* spp. and *Shigella* spp. comprising 38–45% of TD cases.^4^ Contrary to TD, infectious diarrhea and gastroenteritis in the United States is more likely of viral origin.^5,6^ TD can afflict anyone and is one of the most common disease and nonbattle injury ailments in deployed military personnel, with an average incidence of 29% per month, although high rates of 60% or more have been recorded in hyper endemic areas, such as southeast Asia (i.e., Thailand), and early on during operational deployments.^5^ During the last century, despite knowledge of germ theory and efforts toward increasing field sanitation and hygiene measures, the proportionate morbidity of TD as a disease and nonbattle injury has changed very little in deployed military units.^2^

TD in a deployed setting is not merely an inconvenience afflicting military personnel. The development of TD can significantly impact a mission in a deploying unit and result in lost duty time. Median disease duration is reported to be 3 days (interquartile range 2.6, 3.5).^3^ But impacts go beyond days lost. One published report describes an incident in which five of 222 airmen developed diarrhea in one day during a training mission and adversely affected operations.^7^ In an another anecdote of 200 British paratroopers during OPERATION HERRICK 8 (Afghanistan, 2008), each soldier with TD was unfit for duty for at least 8 days (during a 7 month time span) and at one time, 50% of the unit was nonmission capable.^8^ Preventing or reducing lost duty time may provide cost savings to the Department of Defense, and more importantly, increase the effectiveness of deployed U.S. troops in completing their missions. In addition, there can be a long-term impact on individuals with TD; postinfectious functional gastrointestinal disorders may occur with high frequency and could potentially be reduced through TD prevention strategies or by reducing the disease course or severity.^9–11^

In the deployed environment, a systematic review of 52 studies among deployed military and similar populations identified that when afflicted with TD, service members are not likely to seek treatment from a health care provider.^3^ The reasons behind this observation are varied and may include lack of access to care, less severe disease, self-treatment, or a belief that there is nothing to be done to treat the condition.^3^ In addition, in several surveys of health care providers, it has been shown that providers too frequently prescribe only symptomatic treatment and refrain from using antibiotics, which has been shown in multiple randomized controlled trials to shorten the course of disease.^5,12^ Less than 35% of the providers surveyed reported using antibiotics for TD 80% of the time.^5,12,13^ The reasons for this may include a provider’s lack of knowledge that bacterial etiologies account for most cases of TD, the belief that an antibiotic therapeutic regimen must be prescribed for 5–7 days, or confusion about appropriate empiric therapy for TD. Furthermore, studies are emerging to support the finding that loperamide adjuncted single-dose antibiotic regimens are superior to the current and more costly 3-day regimen standard.^14^ Common antibiotic choices for TD are azithromycin and levofloxacin. In head to head studies, both of these prove equally efficacious except in cases of geographic antibiotic resistance patterns.^15,16^

Compared with current practice management, the improvement and application of effective deployment health strategies (increasing health seeking behavior by the individual and improved delivery of effective antibiotic-based therapies) could substantially reduce lost duty time from TD, thereby increasing mission readiness in deployed U.S. troops. The military is seeking other strategies to abate the impact of TD, such as multiplex or pathogen specific vaccines.^17^ In support of the Department of Defense vaccine development rationale and to prioritize vaccines under development, Riddle et al.^17,18^ developed an economic model to evaluate the cost effectiveness of various vaccine research, development, and implementation strategies.

For this study, the economic model previously published was adapted to evaluate the cost effectiveness of implementing improved, evidence-based deployment health guidelines recommending treatment of TD with a single dose antibiotic with loperamide, as well as improving health care seeking behavior (HCSB) among ill deployed personnel.^17^ Scenarios studied were 1) increasing the probability and earlier seeking of care by deployed service members with TD, 2) increasing the probability of military healthcare providers who prescribe a single-dose antibiotic with loperamide for TD, and 3) a combination that includes the previously mentioned strategies. The goals of this study are 3-fold: 1) to describe the cost-effectiveness of increasing healthcare seeking behavior by deployed service members with TD in terms of the cost of averting lost duty time compared with observed (current) health use practices, 2) to describe the cost-effectiveness associated with increasing health care provider use of effective loperamide-adjuncted single-dose antibiotic regimens compared with current use rates and choice of multiday/multidose regimens in treatment TD, and 3) to evaluate the cost-effectiveness of an integrated deployment health management program that considers both improved provider management (single dose antibiotic therapy) and increased healthcare seeking behavior compared with current HCSB and management practices for treatment of TD.

## METHODS

### Model overview.

**Figure 1.**
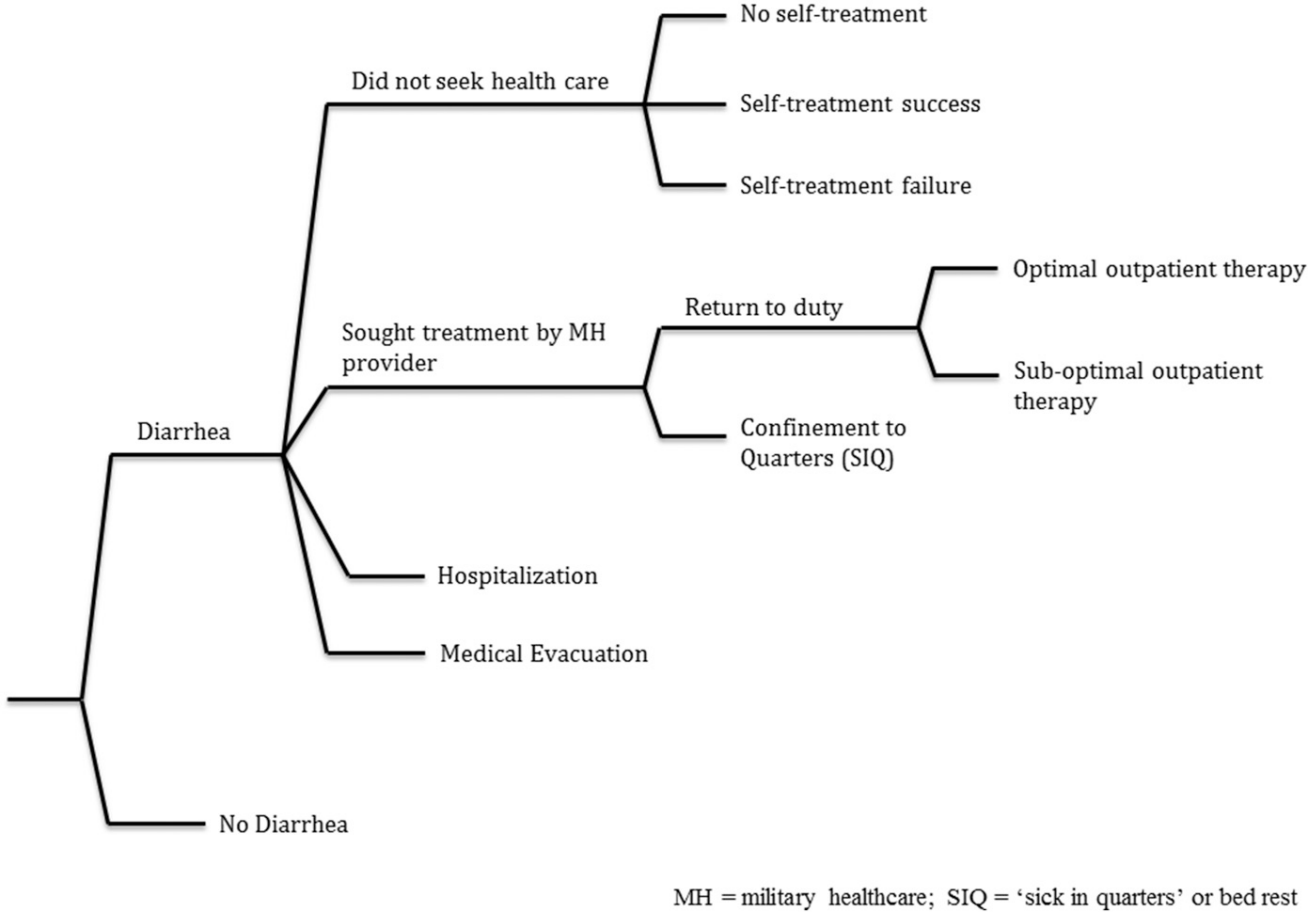
Economic model decision tree. MH = military healthcare; SIQ = sick in quarters (bed rest).

An economic model previously developed to predict the cost-effectiveness of multiplex or pathogen specific TD vaccines was adapted to predict the outcome of the Department of Defense wide revision in treatment standards for TD.^17,19^ Parameters for health care seeking probabilities, costs associated with treatment, and outcomes associated with differential treatment probabilities were used and structured into a cost-of-illness model (Figure 1). The basic cost-of-illness model was built using Microsoft Excel (Microsoft Inc., Redmond, WA) and designed to evaluate changes in health seeking behavior and provider treatment performance to assess how potential changes in education, training, and policy might impact the cost-effectiveness of TD management during deployment. Parameter input categories include the following: 1) deployment size, duration, and incidence of TD during deployment, 2) probability of health care seeking, 3) military healthcare provider treatment preference probabilities, 4) cost of treatment, and 5) duty days lost (DDL) from diarrhea.

For each case of TD, four potential medical dispositions could occur: 1) treatment was provided by a military healthcare provider in an outpatient setting, 2) no treatment was provided by a military healthcare provider, 3) a patient was hospitalized, or 4) a patient was medically evacuated. Within the outpatient treatment branch, either optimal care was delivered, suboptimal care was delivered, or a service member was given bed rest, typically for 24 hours. Optimal care is defined in the base case analysis as any therapeutic plan in which an appropriate antibiotic is prescribed regardless of the dose duration; optimal care is more specifically defined for the *optimized provider prescribing behavior* scenario (described subsequently) as a single dose antibiotic (500 mg azithromycin or 500 mg levofloxacin) with loperamide (4 mg initially followed by 2 mg after each unformed stool, not to exceed 16 mg/day). Single dose treatment with rifaximin was considered but not modeled because of the significant cost difference compared with levofloxacin and azithromycin. Suboptimal care refers to any therapeutic plan that does not include antibiotics.

### Parameter estimates.

Parameter estimates were obtained from a systematic review of incidence, etiology, and impact of TD on deployed military personnel and a previously conducted TD economic analysis.^3,17^ In some cases, expert consensus was used if there was minimal to no published data. Table 1 summarizes the baseline, low, and high estimates, and the used distribution for each parameter. A baseline deployment population was estimated to be 50,000 (range 35,000–80,000) service members which closely represents current operational tempo scenarios (FY2015 estimate).^20,21^ Deployment duration was established at 3.5 months (range 1–12), considerably shorter than a current 12-month deployment in Afghanistan, however, consistent with shorter-term missions and training operations which are the historical norm. Monthly incidence for TD as reported in the systematic review is 28.9% (95% confidence interval 16.2–41.6%). Management probability estimates of TD and outcomes are reported for each decision tree branch.^3,17^ The cost of treatment of each treatment outcome represents the cost to the military health care system and does not include other costs from mission impact or lost personnel time. Cost estimates are provided by a cost-effectiveness analysis for the Department of Defense norovirus vaccination program and updated using the Department of Labor medical cost index for inflation.^18,22^ The upper and lower limits are calculated using a 20% decrease or increase from the base case estimate. The effectiveness of each outcome is reported as DDL from diarrheal illness. The DDL metric encompasses the time spent accessing the latrine and time consumed within the medical system and unable to perform their duties. The average diarrheal illness is characterized as a clinical course of five or more loose or liquid stools per day and persisting for three to four days if left untreated.^23^ From these observations, an assumption is used that time lost to removing and replacing gear, traveling to and from, and using the latrine contributes to a minimum of 6 hours (0.25 days) per diarrheal day experienced in the course of the illness. The systematic review also summarizes the average duration of symptoms before seeking treatment (pretreatment duration) as 1.5 days. Posttreatment duration of symptoms continued for an additional 24 hours on average resulting in 0.125 DDL when optimal care was provided. A detailed description of how these estimates and the other treatment outcome effectiveness estimates were derived is provided by Riddle et al.^17^

**Table 1:** Parameter estimates for base case model

	Baseline	Low	High	Probability distribution
Yearly deployment size	50,000	35,000	80,000	Triangular
Deployment duration (months)	3.5	1.0	12.0	Triangular
Monthly incidence	28.9%	16.2%	41.6%	Normal
Management probability				
[P] of no MH provider rx|illness	69.07%	68.00%	87.00%	Triangular
[P] no self-treatment (run its course)	60.00%	30.00%	90.00%	Triangular
[P] self-treatment success	32.00%	16.00%	48.00%	Triangular
[P] self-treatment failure (seek treatment by MH provider)	8.00%	4.00%	12.00%	Triangular
[P] medical evacuation	0.03%	0.02%	0.04%	Triangular
[P] of treatment by MH provider|illness	30.00%	13.00%	42.00%	Triangular
[P] suboptimal	27.80%	16.00%	51.00%	Triangular
[P] optimal	35.10%	27.00%	57.00%	Triangular
[P] confinement to bed rest	37.10%	13.00%	47.00%	Triangular
[P] hospitalization	0.90%	0.30%	2.40%	Triangular
Cost of treatment type				
Medical evacuation	$16,938	$13,550	$20,326	Triangular
Hospitalization (deployed)	$2,907	$2,325	$3,488	Triangular
Confinement to bed rest	$104	$84	$125	Triangular
Suboptimal	$70	$56	$84	Triangular
Optimal	$82	$65	$98	Triangular
Self-treatment failure	$27	$22	$32	Triangular
Effectiveness outcome (DDL)				
Outpatient (suboptimal)	0.7	0.4	1.0	Normal
Outpatient (optimal)	0.37	0.23	0.52	Normal
Confinement to bed rest	1.6	1.0	2.0	Triangular
Hospitalization	1.7	1.0	3.0	Triangular
Medical evacuation	7	3	10	Triangular
No self-treatment (run its course)	0.37	0.23	0.52	Normal
Self-treatment success	0.18	0.11	0.25	Normal
Self-treatment failure	0.48	0.29	0.67	Normal

DDL = duty days lost from diarrheal illness; MH = military healthcare.

### Management scenarios.

Three TD hypothetical management strategy interventions are presented and compared with the base-case scenario. Table 2 summarizes how the relevant parameter estimates vary between scenarios and degrees of management strategy implementation. The first scenario evaluates the effect of increasing the likelihood that a service member with TD would seek treatment from a healthcare provider while also seeking care earlier in the disease process (summarized as *health care seeking behavior*, or HCSB), in effect reducing the pretreatment duration from 1.5 days to approximately 8 hours (equal to 0.06 DDL). Three different levels of strategy implementation are presented to reflect realistic expectations and achievable goals. To model this scenario, the parameter “[P] of treatment by military healthcare provider|illness” (e.g., the probability of treatment by a military health provider given illness) increased with a compliment decrease in “[P] of no military healthcare provider|illness.” The three levels of implementation modeled increased “[P] of treatment by military healthcare provider|illness” from 30% in the base case scenario to 40%, 55%, and 70%. Resulting from the decreased pretreatment duration, the outcome measure of DDL for optimal treatment and bed rest reduced from 0.37 days to 0.31 days and 1.60 days to 1.41 days, respectively.

**Table 2:** Parameter estimate differences by scenario

	HCSB*			OPB†			Combination		
Parameter (base case)	40%	55%	70%	65%	75%	85%	40%/65%	55%/75%	70%/85%
[P] of treatment by MH provider|illness (30%)	40%	55%	70%	‡	‡	‡	40%	55%	70%
[P] suboptimal (27.8%)	‡	‡	‡	22.0%	15.7%	9.4%	22.0%	15.7%	9.4%
[P] optimal (35.1%)	‡	‡	‡	40.9%	47.2%	53.5%	40.9%	47.2%	53.5%
[P] of no MH provider rx|illness (69.07%)	59.07%	44.07%	29.07%	‡	‡	‡	59.07%	44.07%	29.07%
Optimal treatment cost ($82)	‡	‡	‡	$30	$30	$30	$30	$30	$30
Outcome estimates (DDL)									
Outpatient (optimal) (0.37)	0.25	0.25	0.25	0.33	0.33	0.33	0.08	0.08	0.08
Confinement to bed rest (1.6)	1.38	1.38	1.38	0.88	0.88	0.88	0.66	0.66	0.66

DDL = duty days lost from diarrheal illness.

* Increased health care seeking scenario.

† Optimized provider prescribing behavior scenario, the percentage refers to the ratio optimal:suboptimal.

‡ Value does not change from base case.

The second scenario models the effect of optimizing healthcare provider behavior (summarized as *optimized provider behavior*, or OPB) toward a greater propensity to prescribe a single-dose antibiotic adjuncted with loperamide. The ratio of providers prescribing optimal outpatient care versus suboptimal care is adjusted and modeled at three different levels (from the base case scenario of 55.8%) based on realistic and achievable goals: 65%, 75%, and 85%. Optimal care is prescribed for any patient with TD by means of an algorithmic checklist to enable lower level health care providers, such as medics and corpsmen to provide simple and effective single dose cure therapies, thus reducing the burden of TD on military treatment facilities and reducing the cost of treatment. Because field trials of the described single dose antibiotic therapy demonstrate a reduction in time to last unformed stool to a mean of 15 hours (equating to 0.075 DDL) from 24 hours, DDL decreased from 0.37 to 0.33 days in the optimal treatment branch.^19^ In addition, it is by convention that bed rest is prescribed by a provider typically for 24 hours; however, as informed by field trials for a single dose antibiotic therapy, a 12-hour bed rest period is most appropriate and results in a quicker return to duty of the service member. Therefore, DDL from bed rest reduced from 1.60 to 0.88 days. The cost for optimal care was also adjusted to account for a greater case burden treated by lower level healthcare providers and less through military treatment facilities. Care through a medic or corpsmen was estimated to be $3, primarily from the cost of antibiotics and a course of loperamide and is substantially less that the computed costs when individuals seek care from a deployment treatment facility as defined in the previously developed economic model. Assuming 65% of TD patients would reasonably seek treatment from a medic or corpsmen and 35% would continue to seek treatment from the treatment facility, the cost associated with optimal care was adjusted to $30 per patient.

The third scenario modeled the effects of combining improved HCSB of service members and optimized provider prescribing behavior. As in the previous two scenarios, three levels of implementation were modeled: HCSB increased to 40%, 55%, and 70% with a compliment reduction in the nonhealth care seeking arm and provider use of optimal therapy increased to 65%, 75%, and 85%, respectively. Here, optimal therapy is the same as defined for OPB and result in 0.11 DDL from a pretreatment duration of 8 hours and posttreatment time to last unformed stool (TLUS) of 15 hours.

### Primary outcome measures.

Two primary outcome measures are calculated to compare the relative effectiveness among management strategies at varying degrees of implementation and to the base case analysis. Change in DDL-gained or DDL-averted measures the difference between DDL of the base case analysis and each management strategy scenario independent of cost. The cost effectiveness ratio (CER) was calculated using the formula: (cost difference of scenario from base case)/(DDL-gained or DDL-averted). The CER served to compare the overall cost of a management strategy between each of the scenarios per change in DDL-averted/gained.

### Sensitivity analyses.

Sensitivity analyses were performed to measure the robustness of the model using two techniques. A one-way sensitivity analysis using the SensIt add-on (TreePlan Software, San Francisco, CA) for Microsoft Excel was performed and measured the impact of low and high values for each base case parameter estimate on the base case cost ratio (USD/DDL). The results were reported graphically in a tornado plot to reveal which factors have the greatest effect on base case variability.

A multiple probabilistic sensitivity analysis was also performed to measure the robustness of the model for each scenario using the Monte Carlo simulator SimVoi add-on (TreePlan Software) for Microsoft Excel. In this sensitivity analysis, a random number within a given probability distribution for each parameter estimate is selected and the outcome measure (CER or DDL-averted/gained) is computed. Parameters measured as percentages are normalized so that the cumulative percentage equaled 100%. The CER and DDL-averted/gained were then aggregated from 3,000 iterations and reported as a median value with an interquartile range.

## RESULTS

In a single deployment of 50,000 service members deploying for an average duration of 3.5 months, 50,575 episodes of TD occurred with a management cost to the military healthcare system of $2,974,311 (US dollars, USD). DDL to diarrhea are estimated to be 25,918 (Table 3). When modeled HCSB is increased and afflicted service members seek care within 8 hours of symptom onset, DDL/year increase to 27,101, 30,958, and 34,816 at 40%, 55%, and 70% health care seeking rates, respectively. Each rate level modeled produced rather than averted more DDL (e.g., negative DDL-averted or positive DDL-gained) value: −1,183, −5,041, and −8,898, respectively. The correspondent cost to the military healthcare system was estimated at $3,403,120, $4,046,333, and $4,689,547, respectively. As a result, to represent a net negative outcome to the military health system (increased DDL and increased cost), the CER is expressed $/DDL-gained: $363, $213, $193 per DDL-gained, respectively (Table 3).

**Table 3:** Management scenario outcomes

	Base case	HCSB*			OPB†			Combination		
		40%	55%	70%	65%	75%	85%	40%/65%	55%/75%	70%/85%
Annual episodes	50,575	‡	‡	‡	‡	‡	‡	‡	‡	‡
Scenario total cost	$2,974,311	$3,403,120	$4,046,333	$4,689,547	$2,660,662	$2,622,427	$2,584,192	$2,988,061	$3,405,534	$3,784,775
Cost difference with base case	–	$428,809	$1,072,022	$1,715,236	−$313,649	−$351,884	−$390,118	$13,750	$431,224	$810,465
Duty days lost (DDL)/yr	25,918	27,101	30,958	34,816	21,326	20,973	20,619	19,584	19,590	19,031
DDL-averted	–	−1,183§	−5,041§	−8,898§	4,592	4,945	5,299	6,333	6,328	6,887
Cost ratio ($/DDL)	$115	$126	$130	$134	$125	$125	$125	$152	$174	$199
CER ($/DDL-averted or gained‖)	–	$363‖	$213‖	$193‖	−$68¶	−$71¶	−$74¶	$2	$68	$118

* Increased health care seeking scenario.

† Optimized provider prescribing behavior scenario, the percentage refers to the ratio optimal:suboptimal.

‡ No change from the base case analysis.

§ A negative DDL-averted is equivalent to an increase in DDL, or DDL-gained.

‖ CER = $/DDL-gained in the HCSB scenario to reflect an increase in total DDL and a cost increase.

¶ A negative CER results from a decrease in DDL and an overall cost savings.

Modeling optimized provider prescribing behavior demonstrates cost savings to the military healthcare system with a total cost of $2,660,662, $2,622,427, and $2,584,192 when 65%, 75%, and 85% of providers prescribe optimal outpatient treatment when appropriate, respectively. DDL-averted is 4,592, 4,945, and 5,299, respectively. Representing a decrease in DDL from the base case and an overall cost savings, the CER is estimated at −$68, −$71, and −$74 per DDL-averted, respectively (Table 3).

Combining both management strategies into a model produces a cost estimate to the military healthcare system of $2,988,061, $3,405,534, and $3,784,775 when HCSB increased to 40%, 55%, and 70% with a compliment reduction in the nonhealth care seeking arm and provider use of optimal therapy increased to 65%, 75%, and 85%, respectively. The combination scenario also yielded the greatest number of DDL-averted of 6,333, 6,328, and 6,887, respectively. The resulting CER is $2, $68, and $118 per DDL-averted, respectively (Table 3).

### Sensitivity analysis.

**Figure 2.**
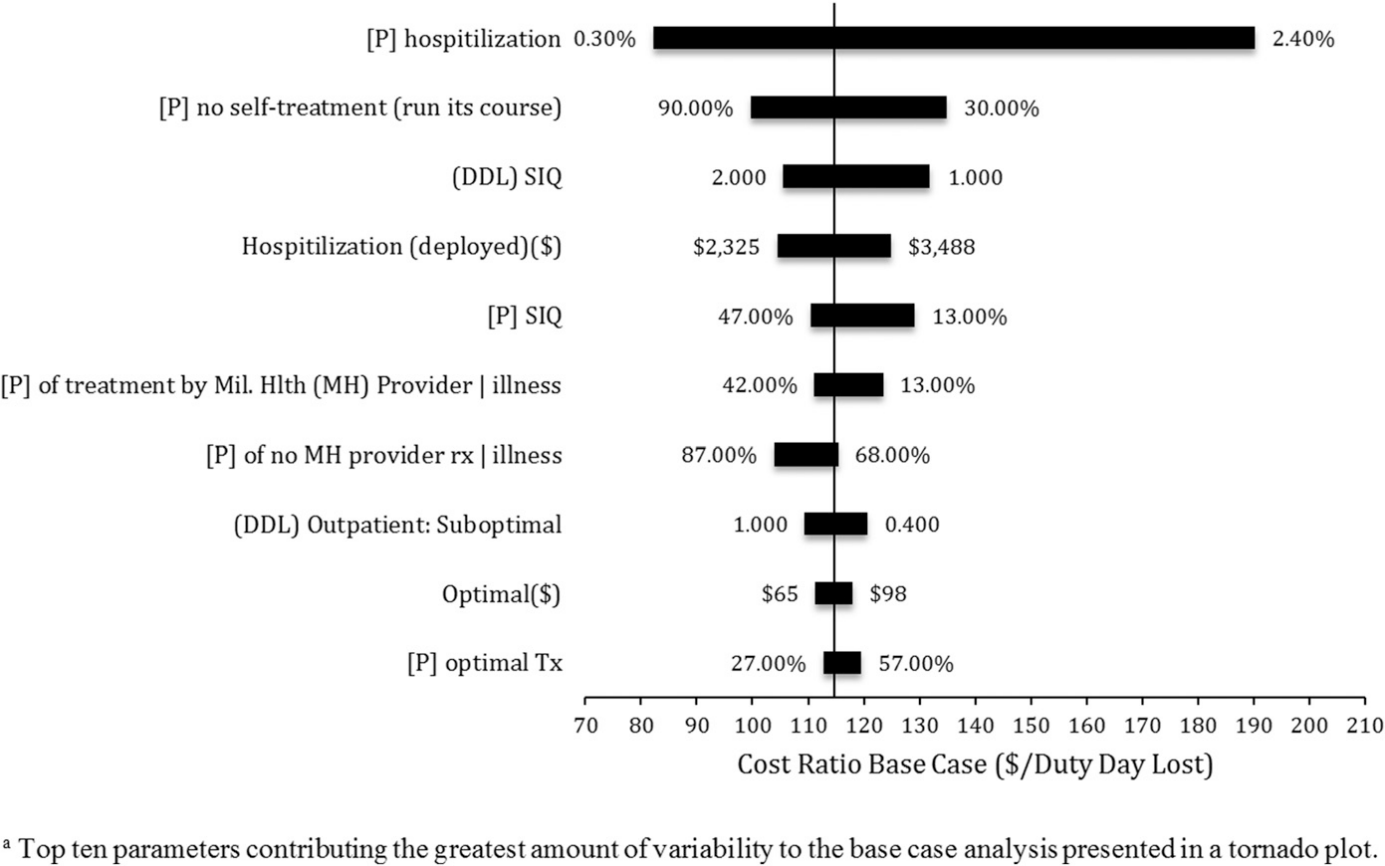
Tornado plot–base case analysis. Top ten parameters contributing the greatest amount of variability to the base case analysis presented in the tornado plot; [P] = probability; DDL = duty days lost; MH = military health; SIQ = sick in quarters (bed rest); Tx = treatment.

The results from the one-way sensitivity analyses are presented in a tornado plot (Figure 2) and include the top ten parameters that contribute to the greatest amount of variability of the baseline CER estimate. The base output value is $114 per DDL. The probability of hospitalization (0.9%) has the greatest effect on the base-case CER variability with a low and high output value estimate of $82 and $190 per DDL, followed by the parameters of “[P] of no self-treatment given no treatment by a military healthcare provider,” “DDL for bed rest,” and the cost associated with hospitalization.

**Figure 3.**
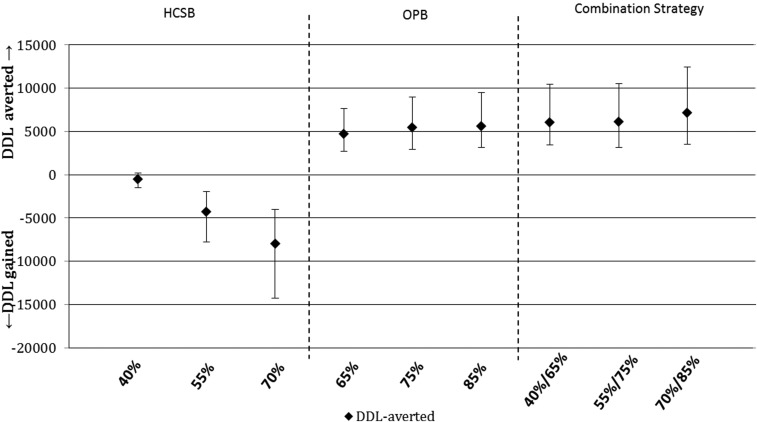
Monte carlo sensitivity analysis–median duty days lost averted with interquartile range. DDL = duty days lost; HCSB = health care seeking behavior; OPB = optimized provider behavior.

**Figure 4.**
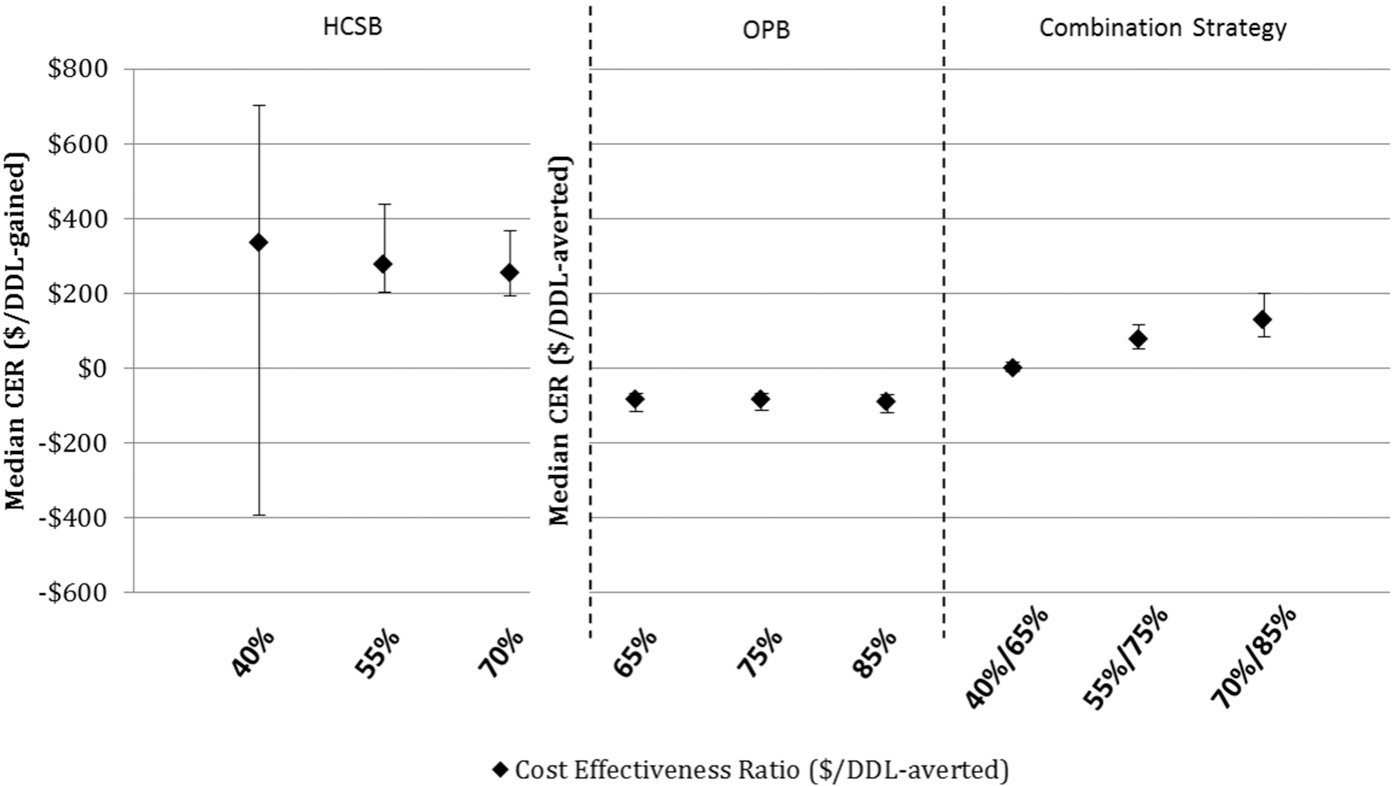
Monte carlo sensitivity analysis–median cost effectiveness ratio with interquartile range. CER = cost effectiveness ratio; DDL = duty days lost; HCSB = health care seeking behavior; OPB = optimized provider behavior.

The multiple probabilistic sensitivity analysis is presented graphically in Figures 3 and 4. The median DDL-averted/gained output metric closely resembled the calculated DDL-averted/gained metric presented in Table 3. Likewise, the median CER output metric also closely resembled the calculated CER. The interquartile range for both DDL-averted/gained and CER is also presented in Figures 3 and 4; with the exception of the CER in increasing HCSB, the interquartile range widens in each scenario with increasing levels of implementation. CER median values and interquartile ranges are presented in Table 4.

**Table 4:** Monte Carlo output measures

	HCSB*			OPB†			Combination		
	40%	55%	70%	65%	75%	85%	40%/65%	55%/75%	70%/85%
DDL-averted									
Median	−512‡	−4,336‡	−7,977‡	4,679	5,402	5,570	6,041	6,111	7,096
IQR	(−1,538, 161)	(−7,781, −1,949)	(−14,257, −4,018)	(2,698, 7,628)	(2,951, 8,963)	(3,151, 9,463)	(3,446, 10,433)	(3,103, 10,532)	(3,532, 12,418)
CER									
Median	$334§	$279§	$253§	−$86‖	−$87‖	−$90‖	$1	$75	$126
IQR	(−$394, $702)	($203, $438)	($192, $367)	(−$115, −$67)	(–$114, −$69)	(−$119, −$70)	(−$11, $15)	($50,$117)	($83, $201)

IQR = interquartile range.

* Increased health care seeking scenario.

† Optimized provider prescribing behavior scenario, the percentage refers to the ratio optimal:suboptimal.

‡ A negative DDL-averted is equivalent to an increase in DDL, or DDL-gained.

§ CER = $/DDL-gained in the HCSB scenario to reflect an increase in total DDL and a cost increase.

‖ A negative CER results from a decrease in DDL and an overall cost savings.

## DISCUSSION

Several interesting findings emerge from this cost effectiveness analysis. This model demonstrates that a deployment Force Health Protection policy implementation designed to increase the likelihood that a service member with TD will access care (without optimal antibiotic-based therapy used) within 8 hours of symptom onset contributes to a greater number of DDL and an overall increase in cost if implemented as a sole management strategy. Essentially, by encouraging more service members with TD to seek care, a greater burden would be placed on the military healthcare system without a commensurate benefit in the improvement of illness outcome. Presently, approximately 30% of service members currently seek care, and 35% are provided optimal therapy. By directing more afflicted service members into the military healthcare system without optimizing treatment protocols, an increase in DDL (DDL) is attributed to additional time spent in the medical treatment facilities without benefit. This result is represented as negative DDL-averted (or DDL-gained). It is important to note that while the DDL parameters are comparable between the “treatment” and “no treatment” arms (0.37 DDL for optimal treatment and allowing TD to “run its course”), other negative outcomes from not seeking treatment must be considered. These include more severe symptoms for a longer duration, a decrease in hygiene and sanitation that may lead to diarrhea in other individuals, and potentially a greater likelihood of postinfectious functional gastrointestinal disorders attributed to longer illness durations.^24^

Optimizing provider-prescribing behavior, as a stand-alone strategy as presented by this model, will result in an overall lower cost to the military healthcare system for TD management while averting up to 5,299 DDL when maximally implemented. Overall, this model demonstrates that this strategy could prove to be cost effective and have a significant impact on medical readiness in deployed service members. The multivariate probabilistic analysis supports this conclusion and demonstrates that even with parametric uncertainty, favorable effectiveness of this strategy would be expected. To practicably achieve these results in a deployed setting, most TD patients would be required to be treated by healthcare providers that are embedded within the operational unit, often in the form of medic or corpsman. Appreciable savings in duty time could be achieved if these healthcare providers were trained and able to follow a simple algorithm which provides appropriate therapy including single antibiotic dose and loperamide; this medical encounter can be accomplished in a matter of 10–15 minutes (approximating empiric self-treatment in a civilian traveler setting) compared with the approximately 1–3 hours spent in a typical deployment medical treatment facility environment.^17^ However, current Department of Defense policy does not allow medics and corpsmen to prescribe antibiotics for this condition.

Furthermore, reducing a standard prescription of bed rest from 24 to 12 hours between reevaluations would contribute a significant savings in DDL. By shifting the burden of TD treatment from the military treatment facility to a unit’s organic healthcare provider (or self-treatment in appropriate scenarios), significant effectiveness could be gained. A training infrastructure already exists through initial entry training of medics and corpsmen, thus making the implementation of this management strategy across the Department of Defense feasible, although a policy change allowing medic/corpsmen the use of antibiotics would be needed.

An integrated Deployment Health TD strategy that encompasses both increased healthcare seeking behavior and optimized provider prescribing behavior is shown by this economic model to contribute to the greatest level of medical readiness. Up to 6,887 DDL could be averted with the combination strategy when maximally implemented as modeled, a 27% reduction. This number is not insignificant when an annual military deployment population of 50,000 is considered. However, compared with the OPB strategy alone, which demonstrated an overall cost savings, the cost of this strategy will increase incrementally parallel to increasing levels of implementation as demonstrated by the increasing CER as modeled (from $2 to $118). While this is the more expensive management strategy presented in this analysis that yields a reduced DDL outcome, this cost is favorable when compared with the FY2015 daily per troop cost of deployment for Operation Enduring Freedom-Afghanistan of $10,800.^25^ Even when compared with lower deployment estimates approaching $700/day using FY2004 data, the combination scenario still represents a cost effective strategy while achieving the greatest level of readiness and unit performance.^17^

Consideration should be given to the required programs, challenges, and likelihood of achieving the optimized strategy changes in this model. Increasing HCSB can be achieved through the predeployment training infrastructure that currently exists so costs could be considered minimal. However, there is an opportunity cost of training time with potential impacts on time devoted to other important training programs. For purposes of this cost-of-illness model, any increase in training costs that this might require was not included in the CER estimates. Provider treatment preference may be more easily influenced through adjustments to clinical practice standards to single dose antibiotic with loperamide regimens. However, the implementation of clinical practice guidelines is also not without costs, although these too are considered negligible as they could be included in current curriculum programs. In addition, pushing down simplified treatment algorithms for TD (and the ability to prescribe antibiotics) to the combat medic or hospital corpsman level could improve both access to care issues, as well as increased optimized therapy. Currently, deployed service members have to access a medical treatment facility where a licensed provider is available who can prescribe antibiotics.

Like all economic models, there are several notable limitations. While there are many sources of published data to inform the parameters in this model, some estimates relied on expert opinion and assumptions. For example, the effect on DDL from diarrheal illness when managed with antibiotic therapy of any duration compared with a single dose antibiotic with loperamide was extrapolated from the TLUS data.^19^ While a single dose antibiotic with loperamide has been established to reduce TLUS, the impact on DDL has not been adequately measured and published. The one-way sensitivity analysis also demonstrates that small uncertainties within each parameter could have a marked impact on the base analysis. The probability of hospitalization was shown to have the greatest impact because hospitalization is associated with the greatest cost of care and the most DDL after medical evacuation. Likewise, the variability in the probability that a service member does not seek care and allows the disease to run its course, DDL from bed rest, the cost of hospitalization, and the probability of bed rest can also have a profound impact on base case analysis and skew the results for each management scenario. This variability calls for more epidemiologic research to be conducted to better understand these parameter estimates, and how a change in healthcare seeking and optimized management affects hospitalization rates to better inform this economic model. An additional limitation is that the results from this analysis represent a global strategy; however, it is well known that regional differences between bacterial prevalence and antibiotic susceptibility exist. In that same light, a strength of this economic model is that it can easily be adapted to specific regions of the world, such as Southeast Asia, where levofloxacin resistance is widespread if the parameters informing antibiotic susceptibility and efficacy are known. A more general limitation to this economic model is its strict focus on the US military in a deployed setting. This model could be adapted to other large organizations operating in similar environments that use a single payer healthcare system and supports pretravel counseling; however, other nation’s militaries may find benefit in this analysis and its recommendations.

This economic model focused on the cost associated with the acute diarrhea and did not consider the impact of long-term sequelae on a service member’s readiness and the additional cost of treatment this might incur. Postinfectious gastrointestinal disorders are well-documented in military populations, and TD is a noted risk factor.^9^ However, it is reasonable to hypothesize that reducing the duration of diarrheal illness may lead to a reduction in postinfectious sequelae.^24^ This would lend to an even greater cost savings to the military health system. Additional research is required to characterize the association between diarrheal illness in which treatment and effect is provided early and postinfectious gastrointestinal disorder incidence.

Finally, the impact of adverse medication events was not considered which could potentially increase the cost of antibiotic treatment therapies. For example, recent data on acquisition of multidrug resistant organisms associated with travel, TD, and the treatment of TD may represent an unrecognized cost, although observations do not describe individual health impacts, and the colonization is transient.^26–28^

This cost effectiveness analysis demonstrates the potential ability to avert DDL in a cost effective manner by implementing a combined strategy of increased (and earlier) access to care and optimized provider treatment use of antibiotics and loperamide. While the cost to the military health system would be higher than the current management practice, the favorable benefits in lost duty days averted for a fraction of the cost to keep a soldier in a deployed environment make a sound trade-off. Deployment Health Guidelines surrounding the management of TD are under development and these data should be useful to those within the Department of Defense who are making decisions about adoption and implementations of these guidelines. The results presented previously suggest several different strategies that could be implemented through policy change and training programs with a positive effect on mission readiness and at a reasonable cost effectiveness.
